# Supplementary material for: “GrimAge,” an epigenetic predictor of mortality, is accelerated in major depressive disorder
Source: Transl Psychiatry. 2021 Apr 6;11:193. doi: 10.1038/s41398-021-01302-0 (PMC8021561; doi:10.1038/s41398-021-01302-0)
Supplement: Supplementary file 1 — Supplementary Material [file 41398_2021_1302_MOESM1_ESM.docx]

**SUPPLEMENTARY MATERIAL**

**Covariance for Blood Cell Composition in AgeAccelGrim**

As an additional quality control to ensure that our results were not the result of idiosyncrasies in blood-cell composition, as the GrimAge metric is sensitive to composition, we repeated our analysis of differences in AgeAccelGrim between MDD and HC by covarying for the percentages of monocytes, neutrophils, lymphocytes, eosinophils, and basophils. The significant effect of MDD status persisted when covarying for blood cell composition only (*F_MDD_(1,95) = 12.773, p=0.001*), and with the addition of current smoking status (*F_MDD_(1,94) = 9.117, p=0.003*) and full adjustment for smoking status, sex, and BMI (*F_MDD_(1,92) = 7.794, p=0.006).*

**Sub-Cohort Analyses of Effect of Smoking on Group Differences in AgeAccelGrim**

We repeated our primary analyses of differences in AgeAccelGrim between MDD and HC in a sub-cohort of participants with detailed data available on lifetime smoking history (41 MDD and 51 HC), such that we could covary for tri-level ‘never,’ ‘former,’ or ‘current’ smoking status instead of binary ‘current’ smoking status. The effect in Models 2 and 3 remained significant when analyzed within this subsample (***Model 6*** *(analogous to Model 2)****:*** *F_MDD_(1,89)=8.310, p=0.005;* ***Model 7*** *(analogous to Model 3)****:*** *F_MDD_(1,87)=6.716, p=0.011)* (**Supplementary Table 1)***.*

While it would be desirable to further assess for differences in AgeAccelGrim among ‘never’ smokers, our sample for such an analysis was too small, with only 20 MDD and 30 HC participants classified as ‘never’ smokers, yielding an observed power of only 0.22 after adjustment for sex and BMI.

**Sub-Cohort Analyses of Effect of Smoking on Group Differences in Raw and Age-Adjusted DNAmPACKYRS**

We similarly repeated our primary analyses of differences in age-adjusted DNAmPACKYRS between MDD and HC in the same sub-cohort. The group difference in age-adjusted DNAmPACKYRS remained significant when analysis was limited to those with available data on lifetime smoking history and covaried for tri-level smoking history (***Model 6:*** *F_MDD_(1,89)=7.386, p=0.008;* ***Model 7:*** *F_MDD_(1,87)=6.818, p=0.011).* We again did not limit our cohort to ‘never’ smokers, as the sample of 20 MDD and 30 HC yielded inadequate power for this *post-hoc* analysis, with an observed power of only 0.27.

These same analyses were repeated for the raw non-age-adjusted DNAmPACKYRS variable. Again we found that the differences between MDD and healthy controls persisted when adjusting for tri-level smoking history (***Model 6:*** *F_MDD_(1,89)=3.968, p=0.049*). However, the group difference was attenuated when we adjusted for tri-level smoking status, sex, and BMI (***Model 7:*** *F_MDD_(1,87)=3.330, p=0.071*).

Of note, there is not to our knowledge consensus in the literature about the need for and utility of age-adjustment in the interpretation of the DNAmPACKYRS variable. Insofar as DNAmPACKYRS was derived to estimate total lifetime smoking exposure, age-adjustment makes the variable difficult to interpret. However, if the DNAmPACKYRS metric is hypothesized to capture elements of aging that are independent of smoking exposure, then an age-adjusted variable may be most relevant. In our sample, we see similar patterns in both the raw and age-adjusted versions of DNAmPACKYRS, with evidence of some attenuation only within a sub-cohort where we covaried for tri-level smoking status, sex, and BMI. However, perhaps this is the result of MDD and healthy control samples that were well-matched on age in our study. Other studies may find discrepancies between the raw and age-adjusted DNAmPACKYRS metrics, and such differences may be informative.

**Effect of Smoking on the cg05575921 Methylation Site, a Component of the DNAmPACKYRS Metric**

Because we identified a statistically significant effect of MDD on the age-adjusted DNAmPACKYRS metric, we considered potential causal mechanisms of the association. One of the methylation sites included in the DNAmPACKYRS metric is the cg05575921 CpG site, which localizes to the Aryl Hydrocarbon Receptor Repressor Gene (AHRR). It has repeatedly been shown that this CpG site demonstrates hypomethylation associated with smoking exposure(1–3), consistent with its selection by machine learning algorithms to the DNAmPACKYRS metric. However, there is also evidence that the AHRR plays a direct causal role in at least some somatic diseases, namely atherosclerosis(4,5), and accumulating evidence for its role in other psychiatric diseases, namely PTSD(6,7). As a result, we sought to assess the relationships between MDD and the cg05575921 methylation site in our cohort.

However, before assessing for disease-specific associations, we first wanted to assess the association of the methylation site with smoking exposure within our sub-cohort with available data on lifetime smoking history (41 MDD and 51 HC). A one-way ANOVA demonstrated a statistically significant effect of lifetime smoking exposure (‘never,’ ‘former,’ ‘current’) on methylation at the cg05575921 site (Brown-Forsythe *F(2,44.68)= 6.374, p=0.004*). Planned contrasts revealed that the effect was largely driven by significant hypomethylation among current smokers (***Never Smokers vs. Any Smoking History:*** *t(49.25)= -2.640, p=0.011;* ***Never vs. Former Smokers:*** *t(42.02)= -0.119, p=0.906;* ***Never vs. Current Smokers:*** *t(17.20)= -3.535, p=0.003;* ***Former vs. Current Smokers:*** *t(27.58)= -2.956, p=0.006).* Among ‘ever’ smokers (defined as the combined ‘former’ and ‘current’ smokers), we also noted a statistically significant negative correlation between the estimated number of lifetime cigarettes and cg05575921 methylation state *(N=42, Spearman Rho= -0.427, p=0.005*). This correlation was at least partially driven by current smokers, as it was attenuated to non-significance when the correlation was restricted to former smokers (*N=28, Spearman Rho= -0.301, p=0.119*). This pattern of findings suggests that, while smoking exposure is associated with hypomethylation at the cg05575921, as has been repeatedly suggested in the literature(1–3), statistically significant differences in methylation state in our study were limited to current smokers. This is in line with a recent paper by McCartney et al., where they examined the epigenetic signatures of smoking in current smokers, and the effects of smoking cessation on DNA methylation in former smokers(8). McCartney et al. report that smoking-related epigenetic changes seemingly require prolonged exposure to cigarette smoke, and are at least partially reversible following cessation(8). To the extent that this is true, our pattern of findings supports the utility of assessing AgeAccelGrim and DNAmPACKYRS among ‘current’ non-smokers when the sample size of ‘never’ smokers is inadequate.

In order to assess the relationship between the AHRR and MDD, as captured by the cg05575921 methylation site, we used an independent samples T-test to assess for differences between MDD and HC in our full cohort and an ANCOVA to assess for differences between MDD and HC after covarying for current smoking status. We did not find statistically significant differences between MDD and HC in cg05575921 methylation state in either the unadjusted or adjusted models.

**Effect of Removing the DNAmPACKYRS and DNAmCystatinC from the GrimAge Algorithm**

To test whether age-adjusted DNAmPACKYRS was driving the significant AgeAccelGrim difference between MDD and controls, we repeated our analysis of group differences in AgeAccelGrim after removing the DNAmPACKYRS component from the GrimAge algorithm as follows: “*AgeAccelGrim_NoDNAmPACKYRS = AgeAccelGrim - 8.3268*0.030398* age-adjusted DNAmPACKYRS.”* This method has been previously described for the same purpose(9). Using this new metric (“AgeAccelGrim_NoDNAmPACKYRS”), the difference between MDD and healthy control participants was attenuated to non-significance *(****Model 1:*** *t(107)=* *-1.534, p=0.128, Cohen’s d=0.30).* Adjusting for smoking, sex and BMI further attenuated any group differences *(****Model 2****: F_MDD_(1,105)=* *2.161, p=0.145;* ***Model 3:*** *F_MDD_(1,103)=* *1.071, p=0.303).* The same method was applied to subtract DNAmCystatinC from the GrimAge algorithm (*“AgeAccelGrim_NoCystatinC = AgeAccelGrim – 8.3268*3.5E-6*age-adjusted DNAmCystatinC)*, and the group effect persisted in all models. However, it should be noted that the DNAmPACKYRS component of GrimAge is weighted much more heavily within the algorithm than DNAmCystatinC, precluding direct comparison.

**References**

1. Philibert RA, Beach SRH, Brody GH. Demethylation of the aryl hydrocarbon receptor repressor as a biomarker for nascent smokers. Epigenetics. 2012 Nov 13;7(11):1331–8.

2. Philibert RA, Beach SRH, Lei M-K, Brody GH. Changes in DNA methylation at the aryl hydrocarbon receptor repressor may be a new biomarker for smoking. Clin Epigenet. 2013 Dec;5(1):19.

3. Zeilinger S, Kühnel B, Klopp N, Baurecht H, Kleinschmidt A, Gieger C, et al. Tobacco Smoking Leads to Extensive Genome-Wide Changes in DNA Methylation. Chen A, editor. PLoS ONE. 2013 May 17;8(5):e63812.

4. Reynolds LM, Wan M, Ding J, Taylor JR, Lohman K, Su D, et al. DNA Methylation of the Aryl Hydrocarbon Receptor Repressor Associations With Cigarette Smoking and Subclinical Atherosclerosis. Circ Cardiovasc Genet. 2015 Oct;8(5):707–16.

5. Cole JW, Xu H. Aryl Hydrocarbon Receptor Repressor Methylation: A Link Between Smoking and Atherosclerosis. Circ Cardiovasc Genet. 2015 Oct;8(5):640–2.

6. the Traumatic Stress Brain Study Group, Logue MW, Miller MW, Wolf EJ, Huber BR, Morrison FG, et al. An epigenome-wide association study of posttraumatic stress disorder in US veterans implicates several new DNA methylation loci. Clin Epigenet. 2020 Dec;12(1):46.

7. Smith AK, Ratanatharathorn A, Maihofer AX, Naviaux RK, Aiello AE, Amstadter AB, et al. Epigenome-wide meta-analysis of PTSD across 10 military and civilian cohorts identifies novel methylation loci [Internet]. Genomics; 2019 Mar [cited 2020 Jul 28]. Available from: http://biorxiv.org/lookup/doi/10.1101/585109

8. McCartney DL, Stevenson AJ, Hillary RF, Walker RM, Bermingham ML, Morris SW, et al. Epigenetic signatures of starting and stopping smoking. EBioMedicine. 2018 Nov;37:214–20.

9. PTSD Systems Biology Consortium, Yang R, Wu GWY, Verhoeven JE, Gautam A, Reus VI, et al. A DNA methylation clock associated with age-related illnesses and mortality is accelerated in men with combat PTSD. Mol Psychiatry [Internet]. 2020 May 7 [cited 2020 Jul 21]; Available from: http://www.nature.com/articles/s41380-020-0755-z
