# Supplementary material for: “GrimAge,” an epigenetic predictor of mortality, is accelerated in major depressive disorder
Source: Transl Psychiatry. 2021 Apr 6;11:193. doi: 10.1038/s41398-021-01302-0 (PMC8021561; doi:10.1038/s41398-021-01302-0)
Supplement: Supplementary file 2 — Supplementary Table 1 [file 41398_2021_1302_MOESM2_ESM.docx]

**Supplementary Table 1: Sub-Cohort Analyses of Effect of Smoking on GrimAge and DNAmPACKYRS**

|  | **Model 6: ^1^** | | **Model 7: ^2^** | |
| --- | --- | --- | --- | --- |
| **MDD vs Control:** | **F_MDD_ (df, df = 1, 89)** | **p-value** | **F_MDD_ (df, df = 1, 87)** | **p-value** |
| Age-Adjusted GrimAge* (aka "AgeAccelGrim") | 8.310 | **0.005** | 6.716 | **0.011** |
| Age-Adjusted DNAmPACKYRS* | 7.386 | **0.008** | 6.818 | **0.011** |
| Non-Age-Adjusted DNAmPACKYRS* | 3.968 | **0.049** | 3.330 | 0.071 |
| p-values reflect 2-tailed significance. All models used age-adjusted metrics of epigenetic age. Age-adjusted metrics were calculated as the residual from regressing GrimAge and its components on chronological age. Age-adjusted GrimAge is denoted as "AgeAccelGrim" to maintain consistency with the literature.  ^1^ Model 6: ANCOVA covaried for trilevel smoking status (Never, Former, Current) (N_MDD_ = 41, N_HC_ = 51) ^2^ Model 7: ANCOVA covaried for trilevel smoking status (Never, Former, Current), sex, and BMI (N_MDD_ = 41, N_HC_ = 51)  * denotes epigenetic age variables that were Blom-transformed to achieve normal distributions. | | | | |
